# PuMA: PubMed gene/cell type-relation Atlas

**DOI:** 10.1186/s12859-025-06236-8

**Published:** 2025-07-29

**Authors:** Lucas Bickmann, Sarah Sandmann, Carolin Walter, Julian Varghese

**Affiliations:** 1 Institute of Medical Data Science, Otto-von-Guericke University Magdeburg, Magdeburg, Germany; 2 Institute of Medical Informatics, University of Münster, Münster, Germany

**Keywords:** Cell type annotation, Database, Machine learning, Natural Language Processing, PubMed

## Abstract

**Background:**

Rapid extraction and visualization of cell-specific gene expression is important for automatic cell type annotation, e.g. in single cell analysis. There is an emerging field in which tools such as curated databases or machine learning methods are used to support cell type annotation. However, complementing approaches to efficiently incorporate the latest knowledge of free-text articles from literature databases, such as PubMed, are understudied.

**Results:**

This work introduces the PubMed Gene/Cell type-Relation Atlas (PuMA) which provides a local, easy-to-use web-interface to facilitate literature-driven cell type annotation. It utilizes a pretrained machine learning based named entity recognition model in order to extract gene and cell type concepts from PubMed, links biomedical ontologies, and suggests gene to cell type relations based on a ranking score. It includes a search tool for genes and cell types, additionally providing an interactive graph visualization for exploring cross-relations. Each result is fully traceable by linking the relevant PubMed articles.

**Conclusions:**

This work enables researchers to analyse and automatize cell type annotation based on PubMed articles. It complements manual curated marker gene databases and enables interactive visualizations. The evaluation shows that PuMA is competitive against an extensive manual curated database across three gold standard datasets and two species—mouse and human. The software framework is freely available and enables regular article imports for incremental knowledge updates.GitLab: https://imigitlab.uni-muenster.de/published/PuMA/

## Introduction

In the field of single-cell RNA-sequencing (scRNA-seq) analysis, the interpretation of the large variety of gene markers, cell types and their specific (co-)expression profiles have been a challenge for many years. Manual annotation is time-consuming and partially subjective [1]. However, as of today, manually curated marker-lists are still used to identify and annotate cells in scRNA-seq analysis. Attempts are made to tackle this problem by databases, such as CellMarker2 (CM2) [2] and PanglaoDB [3]. CM2 is manually curated marker gene database which aims to cover a range of markers based on high quality published articles.

However, these databases are not automatically updated, based on the latest research literature, and extension requires time-consuming manual curation. Therefore, CM2 is limited to covering only 24,591 studies. Similar to CM2, PanglaoDB is a database for exploring and analyzing scRNA-seq data from mouse and human tissues. It contains pre-processed and pre-computed analyses from more than 1054 single-cell experiments, with more than 4 million cells from a wide range of tissues and organs. The authors have also compiled a manually curated list of more than 6000 marker genes, which can be used for cell type annotation. However, this database is also a manually curated snapshot, which ensures high quality, but lacks extensiveness. For instance, the marker genes Slc8a1/Ncx1 [4] and Nppb/BNF [5], which are expressed in mice and are closely associated with cardiac muscle cells, are absent from the CM2 database. Notably, however, these important markers are included in our PuMA database.

Our work, therefore, introduces a natural language processing supported annotation of scientific literature in PubMed to generate an extensive database for gene/cell type relations. It includes a traceable query tool, which ranks the genes or cell types based upon the number of co-occurrences, and links all relevant PubMed entries.

### Related works

Other well-established approaches identifying gene/cell type-relations already exist, some of them aiming at including recent publications. One example is CellMeSH [6], which has been created by processing MEDLINE Medical Subject Headings (MeSH) terms and Gene2Pubmed [7] as resources. However, CellMeSH relies on MeSH-cells, a hierarchy defined by the National Center for Biotechnology Information (NCBI), only covering a subset of PubMed content. Furthermore, it does not provide automatic updates or the corresponding source code. Moreover, MeSH terms are broader and less distinct than its multiple counterparts in the Cell Ontology [8]. The main limitation of CellMeSH is the necessary mapping to the open, accessible, and readily available Cell Ontology database, which requires considerable curation work.

A newly published update to the PubTator [9] uses several artificial intelligence tools to annotate PubMed. However, it has two main drawbacks: (1) it does not disclose the full source code which incorporates the full pipeline, only the used libraries are available, and (2) while the tool is highly beneficial for researching corresponding literature, it cannot fulfil the role for marker based gene/cell type annotation. The results are a list of individual articles, without the necessary relations to cell types. A manual search for articles supporting a relation would require both, a gene and a cell type, making it unsuitable for any automatic relation extraction. It also cannot rank primary cell types based on marker genes, which is unsuitable for automatic annotation.

Other work has therefore outlined the importance and potential of biomedical named entity recognition (NER) for research and discovery [10], which is facilitated through machine learning in the recent years. The process of NER, is the task to extract and classify words inside text, such as gene and cell types. Advancements in the field of natural language processing (NLP) emerged successfully with new deep learning techniques like large language models [11]. While recent advancements have emerged new LLMs, such as ChatGPT and Llama [12], they are either proprietary with substantial costs, or have not been fine-tuned for tasks like Named Entity Recognition, especially in the domain of biomedical texts. In contrast, tools such as MarkerGenie [13] and Advanced Biomedical Entity Recognition and Normalization 2 (BERN2) [14] have been fine-tuned to the domain of biomedical texts. This facilitates the annotation of biomedical entities for large amounts of articles.

In this work, we present PuMA—an on demand updatable gene/cell type-relation database based on PubMed. A locally deployable application provides an easy-to-use interface and interactive graphical visualization of gene/cell type-relations available as a standalone Python script or Docker container, a self-contained environment.

## Implementation

PuMA is a framework that utilizes a widely-used NLP system to annotate PubMed, linking additional identifiers, and ranking the co-occurrences. It is based upon two applications: (1) the updater, which is necessary to integrate new articles from PubMed, and (2) a webserver, used for querying and viewing the results. PuMA annotates abstracts from PubMed and full-text articles from the open PubMed Central (PMC) subset. The application has a two-fold approach: We provide a downloadable database and we also provide the application for updating the database, running independently from our provided infrastructure. The open-source applications are provided as Docker containers, which are self contained environments with all dependencies. The necessary production-database for the updater, and the deployment-database for the webserver, are provided in the same repository. In the following, the database creation and querying are explained in detail.

### Atlas creation scheme

**Fig. 1:**
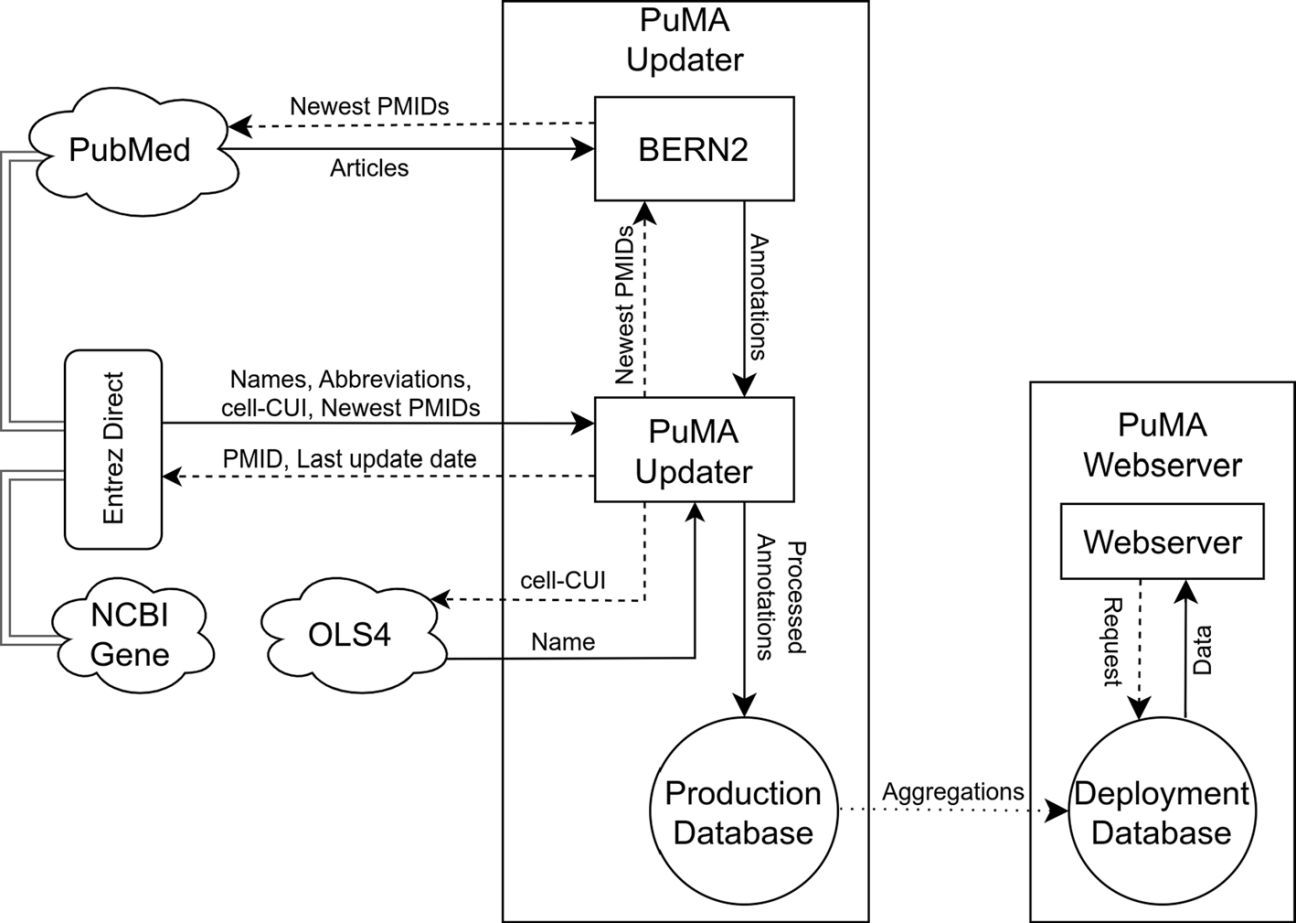
PuMA creation schema, based upon the retrieval databases (left), updater (middle) and the deployable webserver (right). The updater uses BERN2 to annotate PubMed abstracts and full texts, and retrieves additional and standardized information from NCBI Gene and Ontology Lookup Service. The resulting production database is aggregated to and incorporated into the webserver for deployment

The workflow of the annotation and management of PubMed articles involves multiple steps that incorporate various tools and databases to extract, process, and store information about genes and cell types. Initially, the articles are annotated using BERN2, a model specialized in biomedical named entity recognition (NER), which identifies entities like genes and cell types, and maps these to their normalized identifiers (IDs). Annotations for these new articles are computed by multiple BERN2 instances, with concurrent handling of outputs. A filtering process removes annotations which do not map to gene- or cell type-CUIs. The complete workflow is visually summarized in Fig. 1, illustrating the retrieval, processing, updating, and deployment stages across linked databases and resources.

### Mapping of identifiers

These annotations are then processed and filtered to map their IDs to the Cell Ontology (CL) and NCBI Gene [7] databases. For additional meta-information, such as journal names, publication dates, and PMIDs of new articles, the Entrez Direct interface by the National Center for Biotechnology Information (NCBI) [15] is used. Gene concepts unique identifiers (CUIs) are also queried in NCBI Gene to obtain standardized gene names, abbreviations, and the corresponding gene type. The CUIs are maintained by the Unified Medical Language System [16] and helpful to identify common biomedical concepts or to generate semantic core datasets [17]. The European Molecular Biology Laboratory’s Ontology Lookup Service (OLS) in version 4 [18] provides standardized names for annotated cells, to manage spelling inconsistencies across articles.

### Filtering and aggregation

All extracted and standardized information is stored in a production database. This database can be updated regularly, by querying PubMed through Entrez Direct, to fetch the latest PMIDs and articles. The production database, which maintains a historical track of processed articles, undergoes aggregation to generate a deployment database. This aggregation groups genes and cell types by their CUIs, and involves calculating statistics such as the count of distinct PMIDs and total occurrences (see section "Querying and score calculation"). To enhance efficiency, entries with low co-occurrence or fewer referencing articles are filtered.

The deployment database, derived from the production database, is user-friendly and can be updated independently. Users can update it by downloading the latest version, which is provided through a file hosting server. Incremental updates ensure continuity by incorporating new annotations into this streamlined database, while retaining the comprehensive information in the global production database.

#### Querying and score calculation

To query PuMA, genes and cell types should be specified using standardized names from the NCBI Gene and Cell Ontology databases, respectively. Users may query a single gene or a list of genes, which enables automatic annotation with multiple marker genes.

To rank genes or cell types by their relevance or specificity in the literature, PuMA uses a log-normalized scoring metric. This score is designed to consider both: (1) The number of distinct documents in which a gene or cell type appears ($c_{pmid}$) and (2) total number of mentions across all articles ($c_{total}$). These two counts are combined logarithmically (see Eq. 1) to moderate large disparities in frequency and prevent common terms from dominating the ranking. This moderation is particularly important for frequently studied terms like "T-cells," which could otherwise overwhelm less common but still biologically important terms.

1 $$\begin{aligned} score_{raw} = log_{10} (c_{pmids}) * log_{10} (c_{total}). \end{aligned}$$

A widely used method in text mining, term frequency–inverse document frequency (TF–IDF) [19], is commonly applied to highlight rare but potentially distinctive terms. However, in the biological context, TF–IDF tends to down-rank common but important genes or cell types, simply because they appear in many documents; this may exclude essential and well-studied entities from the top results.

**Fig. 2:**
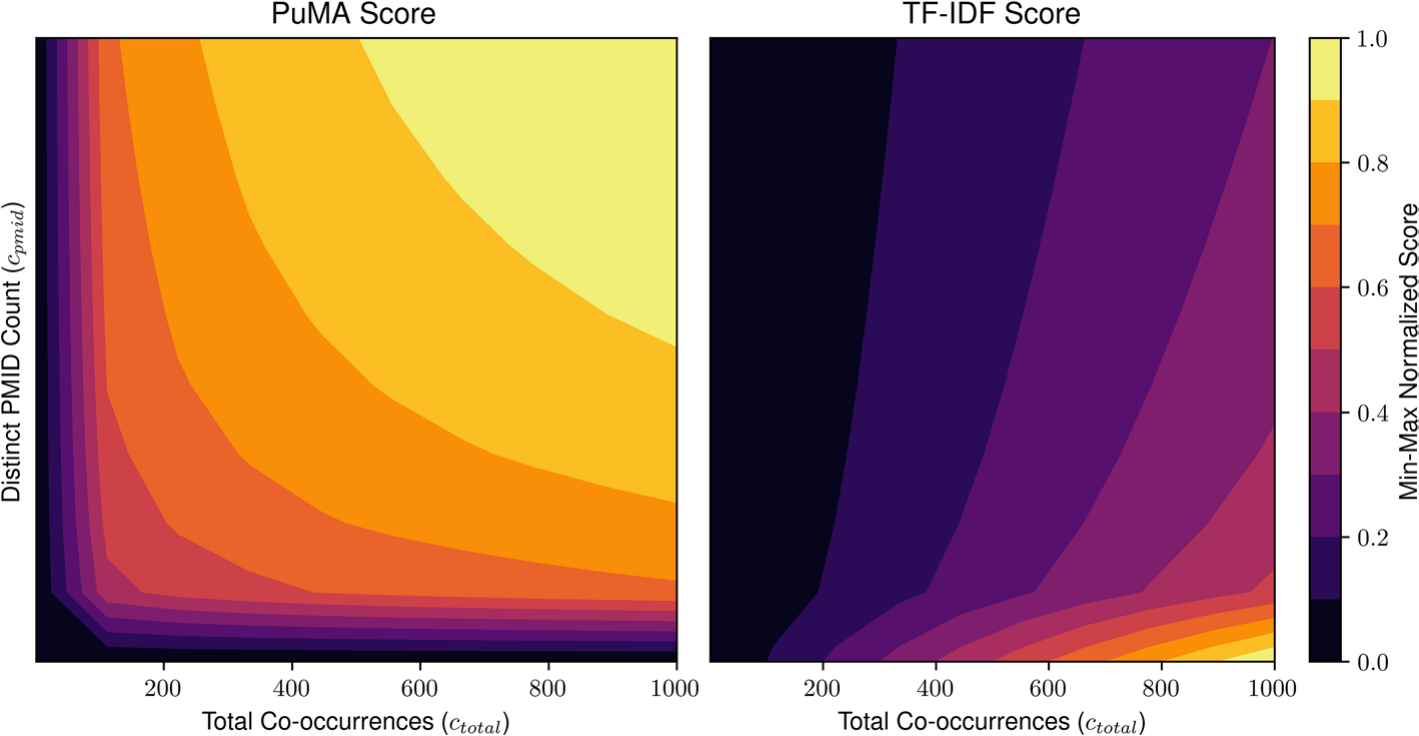
Comparison of the PuMA score (Eq. 1) versus aggregated TF–IDF, plotted against total co-occurrences ($c_{total}$) and the number of distinct PMIDs ($c_{pmid}$)

Unlike TF–IDF, the PuMA score uses logarithmic scaling on both frequency measures to preserve the overall distribution and ensure commonly studied yet biologically relevant terms retain proper significance in the results. Figure 2 shows how the PuMA score relates to aggregated TF–IDF values across various document frequencies, with scores normalized for an example corpus [(D = 195,288) documents].

In summary, while TF–IDF penalizes frequently mentioned terms—potentially overlooking well-established biological entities—the PuMA score more equitably evaluates all genes and cell types by balancing document spread and total mentions. This approach has demonstrated greater robustness and efficiency in internal tests, making it well-suited for annotating large lists of marker genes.

#### Webserver

The webserver operates as an independent Docker container, offering all essential interfaces for a web application to interact with the database. These interfaces include a search field, an exact match filter, a gene type filter, and a cutoff parameter for low-scoring results, among others. User queries, such as those for genes or cells, are processed based on front-end requests, the query term, and search parameters. A SQL query is executed on the deployment database, and the results are aggregated based on the retrieved PMIDs, followed by the calculation of scores for each entry.

Additional functionalities have been implemented, including searching, sorting, and exporting the table. All relevant PubMed articles for each result are listed and linked in a separate interface, enhancing transparency and the explainability of the results. The listed PubMed IDs are interactive buttons that link to their corresponding PubMed entries and open each article in a new tab. This design results in a highly traceable tool, where all referenced articles are linked for the user, and can be accessed individually for each hit. Table 1 provides an example of a gene search result for the term "A1BG", sorted by descending score.

**Table 1:** Extended database result for search-term "alpha-1-B glycoprotein"

Cell type	$c_{pmid}$	$c_{total}$	Score
B cell	110	222	4.79
Platelet	64	204	4.17
Enucleate erythrocyte	67	188	4.15
T cell	73	129	3.93
Neutrophil	35	80	2.94

The table is sorted by descending score, showing the full cell type name, the number of referencing PubMed IDs, all co-occurrences in these articles, and the corresponding score

#### Graphical representation of gene and cell type interactions

The graphical model is a visualization tool designed to explore and analyze interactions between genes and cell types based on their co-occurrences in PubMed articles. By representing genes and cell types as nodes and their co-occurrence relationships as edges, users can visually interpret the frequency and strength of these interactions.

The graph is generated by querying the deployment database with specific search terms relevant to both genes and cell types. The results of these concurrent queries are merged to create a cohesive graphical representation. In this graph, each node size and edge width are scaled according to their scores, reflecting the strength of evidence or frequency of co-occurrence. Using the Compound Spring Embedder Layout (CoSE) [20], a force-directed graph layout, the visualization achieves an organized and coherent representation of complex networks. Nodes representing genes are labeled with abbreviations to ensure clarity and readability, while additional details — such as full gene names— can be accessed by selecting individual nodes.

**Fig. 3:**
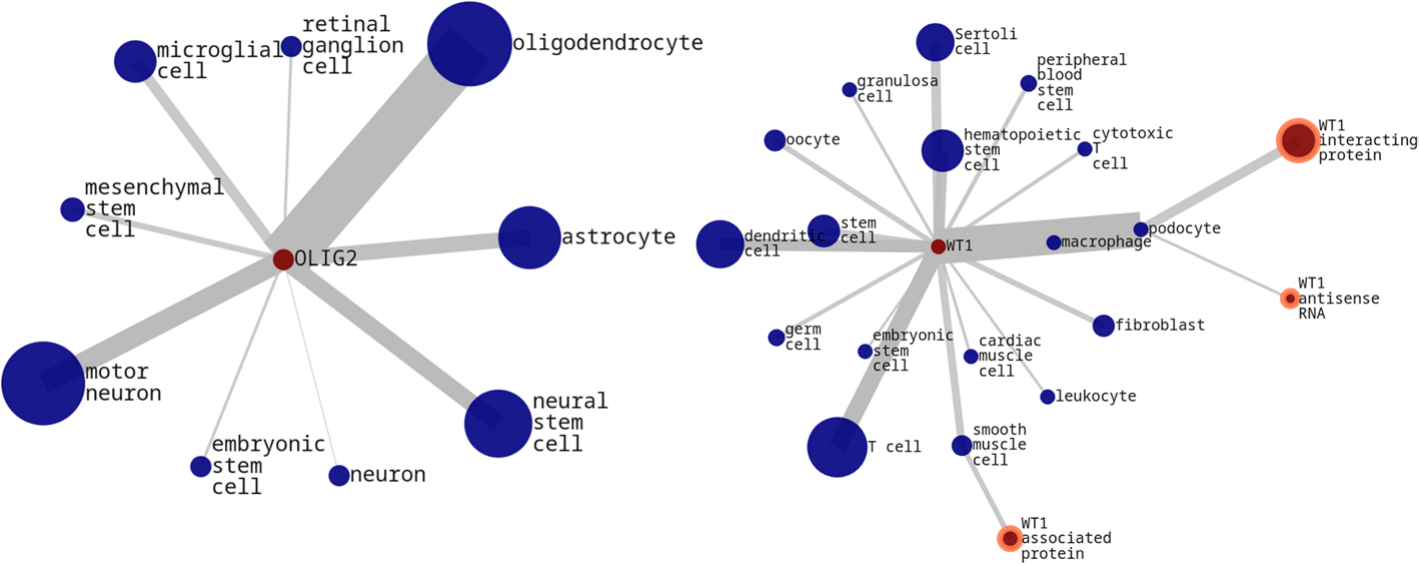
Example graphs for the search-term "OLIG2" (left) and "WT1" (right). Genes and cell types are represented by red and blue circles respectively. Nodes and the width of edges are scaled by their score. Selected genes are highlighted (orange outline) and show the full gene name

Example visualizations in Fig. 3, based on the search terms "OLIG2" and "WT1", highlight the diversity and specificity of gene and cell type interactions. For instance, the "Oligodendrocyte transcription factor" (OLIG2) demonstrates a stronger association with "oligodendrocytes" compared to other cell types like "astrocytes" or "neural stem cells". Similarly, interactions involving the "Wilms’ tumor gene 1" indicate connections with "WT1 antisense RNA" and "WT1 interacting protein" in "podocytes".

#### Application interface

The tool also offers an API endpoint specifically for Python projects, enabling easy access to the same database through a Python class and interface. It provides the same functionality, except for graphical visualization and the combined query used for graph searches. This API can be conveniently installed via a Python package manager.

### Evaluation

We conducted an evaluation of query results using three gold standard scRNA-seq datasets with known cell types. These cover two mouse datasets: Tabula Muris (TM) [21], and the Mouse Cell Atlas [22]. Additionally, we include the human Peripheral Blood Mononuclear Cells (PBMC) dataset [23]. This evaluation was performed in comparison to CM2, a manually curated database that includes experimentally supported markers of various cell types. The comparison was made against a gold standard database to ensure high-quality results. The selected scRNA-seq datasets are well-characterized, publicly available, and represent two relevant species—mouse and human.

CM2 was chosen for this evaluation because it is one of the largest and most up-to-date curated databases available. It contains selected and extracted gene/cell type relations but does not necessarily include all possible relations. Therefore, an evaluation can only be based upon recall, and cannot differentiate in terms of precision. Additionally, the evaluation is based on the primary use of these databases, to extract the cell type based upon found markergenes, regardless of their supporting articles.

For accurate comparisons, we applied filtering to both CM2 and PuMA databases. Species filtering was applied to CM2 to increase its sensitivity, focusing selectively on human and mouse data respectively. For PuMA, only gene/cell type relations mentioned by at least three distinct articles each were retained in the deployment database, reducing the false positive rate and increasing accuracy. No species filtering was applied to PuMA, as internal testing showed no relevant accuracy gain, however marginal computational overhead.

#### Marker gene selection and cell type scoring

We conducted an evaluation to compare two approaches, focusing on calculating a unified distance score for each of the highest differentially expressed genes (DEGs) identified by Mao et al. [6]. This study originally used gene expression clusters and reference cell type annotations from the Tabula Muris (TM), the Mouse Cell Atlas (MCA) and Peripheral Blood Mononuclear Cells (PBMC) datasets. For each cluster, the top $n=50$ DEGs were extracted based on a 1-versus-rest gene expression ratio. We chose 50 genes per cell type, which are considered marker genes, as this number is specific enough for a broad analysis, and higher than manual analysis could provide in a reasonable time window. In our analysis, we examined the distance scores (see section "Distance score") through a top-1 hit per gene evaluation. First, a search for each distinct DEG within both databases to identify any cell type match is conducted. Any cell type hit corresponds as a matched gene. Evaluating the distance involved assessing how closely the top-1 matched gene/cell type relation aligned with the annotated reference cell type. The results were visualized by plotting the mean difference across all genes, complete with a 1 sigma confidence interval (68%) to account for varying gene performance.

We also assessed the combined performance by determining the lower top-1 hit distance from either database for each gene. By combining CM2 with PuMA, our automatically generated extensive database, we anticipate a substantial increase in the number of found genes and cell types. In practice, we query both databases for the same marker genes and use the combined minimum distance for each result. If a result is exclusive to one database, we rely on its singular top-1 hit. This approach enables us to explore the benefits of integrating curated and comprehensive databases, to improve the accuracy and breadth of gene and cell type identification.

#### Distance score

**Fig. 4:**
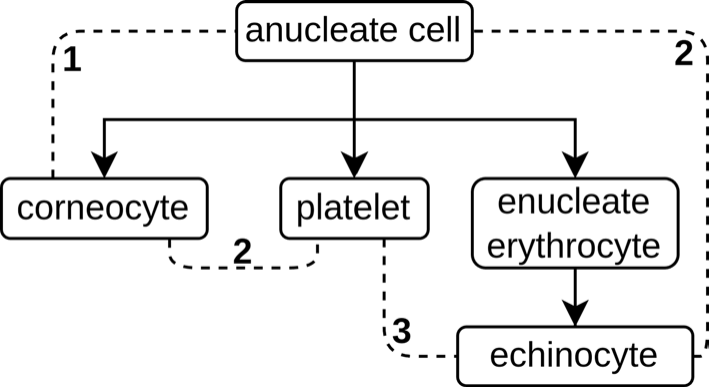
Example of a Cell Ontology graph with super- and subclasses. The dashed lines represent the distance scores between cell types. The distance score is a value which represents the relatedness between cell types

We assess the "correctness" of annotated versus reference cell types from scRNA-seq datasets using a standardized scoring system based on the Cell Ontology database. Utilizing the v2023-07-20 release, we navigate its directed acyclic graph with a modified Dijkstra algorithm [24]. Figure 4 shows an example annotation: Parent and child relationships, such as between "anucleate cell" and "corneocyte", increase the score by one, while sibling relationships, like those between "corneocyte" and "platelet", increase the score by two. A perfect score of zero indicates that the nodes are identical. This scoring system reflects the biological hierarchy that superclasses tend to be more reliable and share more commonalities than specialized subclasses that are only partially related. This method provides a structured way to evaluate the alignment between annotated and reference cell types, ensuring accuracy and reliability in their identification.

## Results

We compare the results of CM2, a manually curated gene/cell type database, with this work, PuMA. This includes the amount of found genes, and the comparison between corresponding suggested cell type to the reference cell type using three gold standard datasets—Tabula Muris (TM), Mouse Cell Atlas (MCA) and human Peripheral Blood Mononuclear Cells (PBMC).

### Database Sizes

Table 2 shows a comparison of contained annotations in CM2 and PuMA (snapshot of 18.01.2024).

**Table 2:** Comparison of entries in CM2, and the deployment database of PuMA (snapshot of 18.01.2024)

Entry	Subtable	CM2	PuMA
Distinct cells	Total	449	1 009
	Co-occurences	$\sim$	628
Distinct genes	Total	22 037	49 336
	Co-occurences	$\sim$	19 615
Co-occurences	total	96 074	195 288
Annotated articles	Total	6 322	9 847 891
	Co-occurences	$\sim$	320 282

Co-occurences for cells and genes are corresponding entries which have at least one gene/cell type-relation. The total annotated articles only includes PubMed articles with at least one gene or cell type

PuMA contains the highest number of distinct cells (increase by 2.25x) as well as distinct genes (factor 2.24x) in comparison to the CM2 database. The total number of found co-occurences is larger than CM2 for cell types (1.39x), and lower for genes (0.89x). The total number co-occurences increases manifold (2.03x), as well as the total number of annotated articles, with at least one gene or cell type, by 1557.72x. This outlines its size advantage compared to CM2 for cell types and general article support. Note that this only includes the relevant processed articles with filtered annotations; the total screening number differs substantially. The applied filtering of PuMA is performed before being deployed to the user. Therefore, the total number of cells and genes annotated is even higher, and additional articles may influence a broad variety of yet to be included co-occurrences.

**Table 3:** Matched number of top 50 Differential Expressed Genes in their respective databases for Tabula Muris, Mouse Cell Atlas and Peripheral Blood Mononuclear Cells

Dataset	PuMA	Both databases	CM2	Neither database
Tabula Muris	134	1619	757	240
Mouse Cell Atlas	548	3129	375	7008
PBMC	274	132	4	90
Total	956	4880	1136	7338

Table 3 outlines the occurrence of genes in either database for the gold standard datasets. For reference, 274 additional genes are found in PuMA, but not in CM2 for the PBMC dataset. This corresponds to $\approx$ 75% (274/(274+90)) of previously unannotated genes by CM2. For the TM dataset, our database extends CM2 by $\approx$ 36% (134/(134+240)) of previously unannotated genes. The MCA dataset is extended by $\approx$ 7.25% (548/(548+7008)).

### Annotation performance

**Fig. 5:**
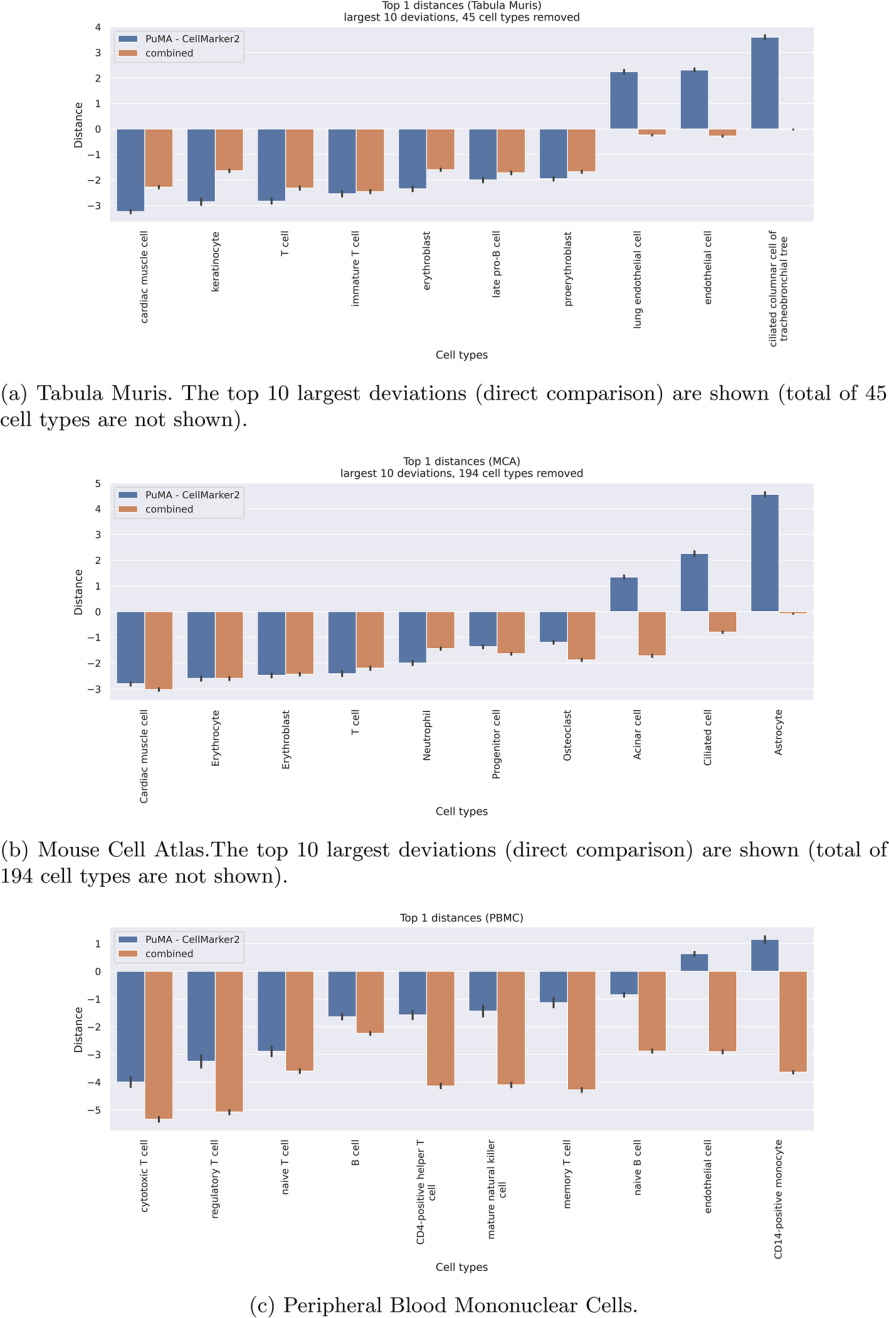

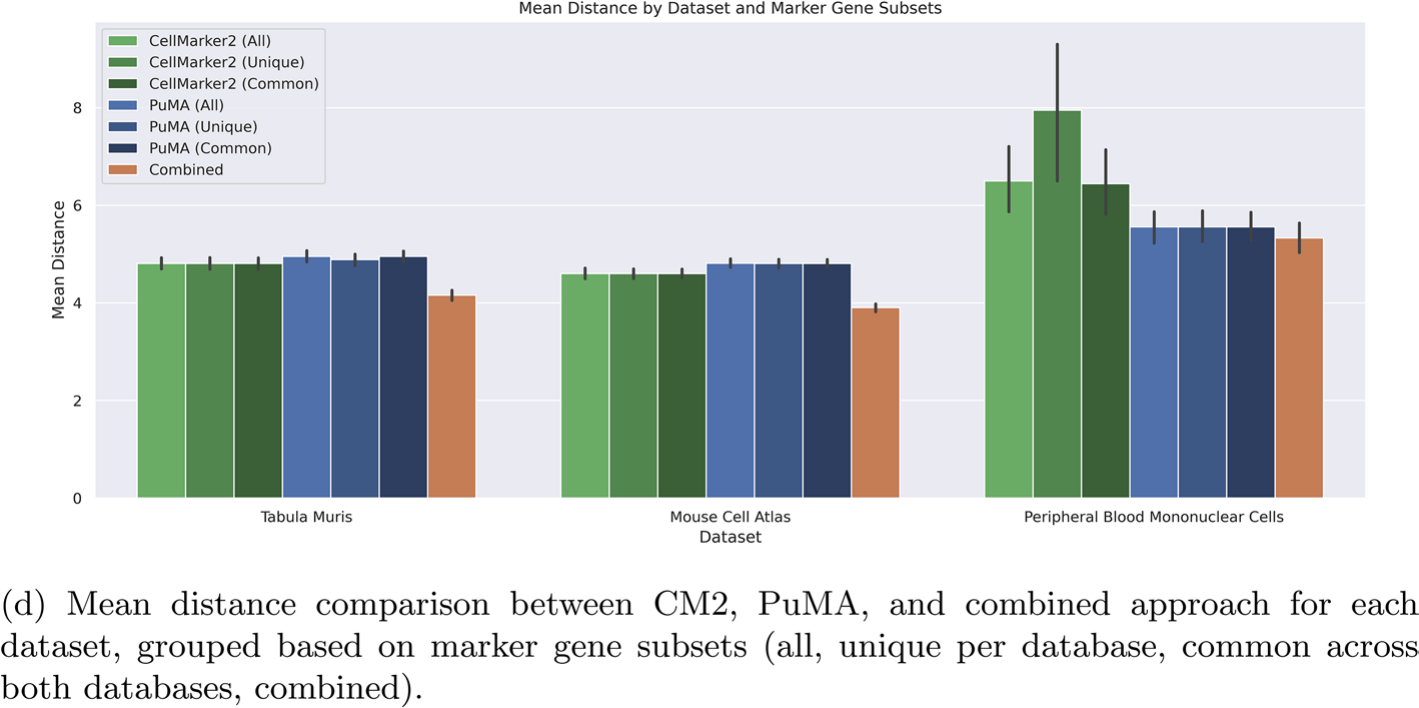
Per cell type distance performance for all genes (top-1 hits) for TM (**a**), MCA (**b**), and PBMC (**c**). The x-axis denotes all cell types, while the y-axis shows the distance. Lower values indicate better annotation performance of PuMA, while higher values suggest better performance of CM2. The combined distance shows the combined average distance reduction for reference cell types by using the minimum distance of PuMA or CM2 respectively, in comparison to CM2. **a** Shows the overall assessment across the datasets without reference normalisation

In Fig. 5, we present a performance comparison between PuMA and CM2 on the TM, MCA and PBMC datasets. The plot highlights the mean and confidence interval for each cell type, with a distance metric where a score of 0 indicates equal performance, a score of 1 suggests a difference akin to super/sub-type, and a score of 2 is comparable to a sibling node (see sections "Marker gene selection and cell type scoring" and "Distance score" for further explanation). Additionally, the combined distance is visualized, which represents the minimal distance of the top-1 hit across both databases for all matched genes and cell types.

2 $$\begin{aligned} \begin{gathered} combined = min(PuMA, CM2). \end{gathered} \end{aligned}$$

PuMA consistently outperforms CM2 across a variety of cell types and maintains a competitive edge across the datasets (see Fig. 5a–c). Although CM2 shows stronger results for some cell types within the TM and MCA dataset, PuMA equals or surpasses the top-1 performance of CM2 of matched genes in over 62.26% in TM, 59.45% in MCA, and 79.55% in the PBMC dataset. When examining directly comparable overlapping genes, PuMA achieves a mean distance reduction of approximately $-$1.51 in the PBMC dataset. While there is a slight increase of +0.0389 in the TM dataset, and +0.1833 in MCA, PuMA still shows robust performance overall. For all datasets, the top-10 largest deviations are in favor for PuMA for 7 out of 10 cases in TM and MCA, and 8 out of 10 for PBMC. For the latter, the combined distance is exceptionally lower, due to marker genes which are being complimented based on both datasets.

Figure 5d shows the distance comparison across subset of marker genes for each dataset. The differences between all, unqiue and common marker genes across both databases are in between error-margins for the TM and MCA dataset. For PBMC and the corresponding unqiue marker genes, the distance is higher for CM2 than for the other subsets. However, these only includes four marker genes, showing high statistical variability. Considering all marker genes, PuMA demonstrates a median distance compared to CM2, with scores of +0.145 in TM, +0.213 in MCA, and $-$0.941 in PBMC. This indicates that PuMA performs well in the PBMC dataset, and is nearly on par, but slightly worse, with CM2 in the TM and MCA dataset. The combined mean distance across all marker genes is lower for all datasets.

## Discussion

In this work, we presented PuMA, a novel, fully traceable database for gene/cell type-relations, which can automatically process PubMed articles, to include recent literature. The web-based application provides a variety of tools, such as marker gene or cell type search and rapid graph-based visualization. Particularly cross-interactions between marker genes and different cell types can be interactively explored using PuMA. This may uncover previously overshadowed relations and empowers researchers to subsequently search for any related interactions. Additionally, we provide the user with all relevant articles to each result, which leads to an explainable tool with high traceability.

The compared commonly used datasets are widely spread and recognized. The evaluation is based on the given reference cell types and marker genes from the corresponding publications. We cannot fully ensure that biological truth is fully represented for all cell types, and that the used clusters and its derived genes are completely distinct marker genes and there is no wrong clustering involved. Still, the evaluation is the same for PuMA and CM2 and does not suffer from any advantage for either of these datasets. While our database shows comparable performance for complete datasets, the quality varies on a gene-by-gene basis. Still, we can reach competitive performance across two datasets and even outperforming a manual curated database in the PBMC dataset. Meanwhile, PuMA also provides additional easy-to-use tools to biologists. While CM2 has a website available, it is hosted on an unencrypted server, in contrary to our solution. Additionally, users are reliant on the server infrastructure to use the additional tools. While we provide a website, we also provide locally deployable containers based on Docker, not only for the web-interface, but also to update the dataset. This additional installation may hinder users from install it locally. However, we included detailed instructions to the user to provide them with necessary basic knowledge for a local installation.

Based on our results, we showed that PuMA is competitive against an extensive, up-to-date curated database, while extending the possible cell types. In addition, we show that a combination of PuMA and CM2 does bring incremental improvements, with a mean distance reduction regarding cell type similarity for three datasets at approximately 14.70%,17.39% and 34.39% respectively, while being very competitive across three datasets and two species. Since PuMA can incorporate additional articles, we expect its performance to improve further in the next few years while new relevant articles are published on PubMed.

## Conclusion

PuMA is a research tool for generating and visualizing gene and cell type relations by utilizing a large language model and the PubMed database. The explainability is facilitated through automated processing and linking to PubMed articles. PuMA performs competitively against a manually curated and up-to-date cell-marker database across three datasets and two species. The combined approach of a high-quality manually curated database and PuMA extends matching genes and improves the overall annotation performance.

## Data Availability

The datasets generated and/or analysed during the current study are available in the Bioinformatics repository, https://doi.org/10.1093/bioinformatics/btab834 [6]. The datasets generated and/or analysed during the current study are available in the GitLab repository, https://imigitlab.uni-muenster.de/published/PuMA.

## Abbreviations

PuMA: PubMed Atlas
PMC: PubMed Central
CM2: CellMarker2
BERN2: Advanced biomedical entity recognition and normalization
OLS: Ontology lookup service
NCBI: National Center for Biotechnology Information
CL: Cell ontology
NER: Named entity recogniotion
CUI: Concepts unique identifier
PMID: PubMed identifier
TF–IDF: Term frequency–inverse document frequency
DEG: Differential expressed gene
TM: Tabula muris
PBMC: Peripheral blood mononuclear cells
MCA: Mouse Cell Atlas
